# Assessment of patient safety challenges and electronic occurrence variance reporting (e-OVR) barriers facing physicians and nurses in the emergency department: a cross sectional study

**DOI:** 10.1186/s12873-020-00391-2

**Published:** 2020-12-14

**Authors:** Ahmed I. Albarrak, Ammar S. Almansour, Ali A. Alzahrani, Abdulaziz H. Almalki, Abdulrahman A. Alshehri, Rafiuddin Mohammed

**Affiliations:** 1 Medical Informatics Unit, Medical Education Department, Research Chair for Health Informatics and Promotion, College of Medicine, King Saud University, P O Box 63709, Riyadh, 11526 Saudi Arabia; 2 College of Medicine, King Saud University, Riyadh, Saudi Arabia; 3 Department of Health Informatics, College of Health Sciences, Saudi Electronic University, Riyadh, Saudi Arabia

**Keywords:** Electronic occurrence variance reporting, Emergency department, Healthcare, Patient safety, Barriers to reporting, Physicians, Nurses

## Abstract

**Background:**

The purpose of patient safety is to prevent harm occurring in the healthcare system. Patient safety is improved by the use of a reporting system in which healthcare workers can document and learn from incidents, and thus prevent potential medical errors. The present study aimed to determine patient safety challenges facing clinicians (physicians and nurses) in emergency medicine and to assess barriers to using e-OVR (electronic occurrence variance reporting).

**Methods:**

This cross-sectional study involved physicians and nurses in the emergency department (ED) at King Khalid University Hospital (KKUH) in Riyadh, Saudi Arabia. Using convenience sampling, a self-administered questionnaire was distributed to 294 clinicians working in the ED. The questionnaire consisted of items pertaining to patient safety and e-OVR usability. Data were analyzed using frequencies, means, and percentages, and the chi-square test was used for comparison.

**Results:**

A total of 197 participants completed the questionnaire (67% response rate) of which 48 were physicians (24%) and 149 nurses (76%). Only 39% of participants thought that there was enough staff to handle work in the ED. Roughly half (48%) of participants spoke up when something negatively affected patient safety, and 61% admitted that they sometimes missed important patient care information during shift changes. Two-thirds (66%) of the participants reported experiencing violence. Regarding e-OVR, 31% of participants found reporting to be time consuming. Most (85%) participants agreed that e-OVR training regarding knowledge and skills was sufficient. Physicians reported lower knowledge levels regarding how to access (46%) and how to use (44%) e-OVR compared to nurses (98 and 95%, respectively; *p* < 0.01). Less than a quarter of the staff did not receive timely feedback after reporting. Regarding overall satisfaction with e-OVR, only 25% of physicians were generally satisfied compared to nearly half (52%) of nurses.

**Conclusion:**

Although patient safety is well emphasized in clinical practice, especially in the ED, many factors hinder patient safety. More awareness is needed to eliminate violence and to emphasize the needs of additional staff in the ED. Electronic reporting and documentation of incidents should be well supported by continuous staff training, help, and feedback.

## Introduction

Patient safety is a major challenge faced by healthcare professionals (HCPs) in hospitals. Patient safety is defined as an impeding and reducing of unfavorable consequences or injuries arising from the processes of healthcare [1].

Healthcare is a complex system that contains many risky processes and requires considerable effort among professionals. The ultimate purpose of patient safety is to avert and lessen the chances of injury, errors**,** and harm that could occur during the provision of healthcare services. Patient safety is improved by leadership, commitment, documentation, and using a reporting system by workers to learn to prevent potential errors [2].

Furthermore, mistakes are opportunities to learn from and can help improve patient safety [3].

Emergency departments (EDs) in hospitals are critical and high-risk environments due to the urgency of care needs and the complexity of communication [2, 4]. The ED is inherently vulnerable to errors that can result in patient harm [5]. A clinician working in the ED encounters many challenges such as overcrowding, poor communication, violence**,** and pressure at work [5, 6]. These conditions could be due to a high number of patients, limited inpatient beds, insufficient medical staff, lack of equipment or lack of knowledge about how to use it, or broken equipment, or a late reply from specialists for a consultation [2, 5, 7, 8]**.** ED workers are more liable to encounter violence compared to other hospital employees since they are in the front line of services to patients [9]. Previous studies have shown that the rate of underreported violence is high and that most of the reports are about major physical injury [6, 10, 11]. Patient safety incidents also have emotional, psychological, social**,** and economic consequences for the families involved, and for healthcare staff [12].

These challenges can be avoided or managed by implementing an incident reporting system such as electronic occurrence variance reporting (e-OVR) to gather and document information about patient safety incidents, which is an important element in the enhancement of patient safety [13–15]. An incident reporting system is necessary to secure patients’ as well as staff safety, indispensable care, and organizational risk management. “Variance” is a measure of something that is not compatible with the standard or regular course of procedures of the hospital organization among its staff and in terms of patient care [2]. A very important step in minimizing adverse events is to ensure prompt reporting among healthcare providers in order to identify the reasons for the variance and to use errors as an educational opportunity and opportunity for quality improvement [1, 16]. Regrettably, there are many barriers preventing clinicians from using e-OVR, such as time pressures, unclear processes, systems not providing confidentiality, lack of feedback, pressure from colleagues, fear of job loss or other punishment, complexity of the reporting system, lack of instructions, work pressure, forgetfulness, and minor errors [17–22].

To ensure successful implementation of strategies to create a culture of patient safety, health care providers require policies, effective leadership, clear guidelines, and direction to drive safety improvements and create adequate confidence among patients in their care. Over the past few years, the use of incident documentation systems has become widespread in many hospitals. However, an extensive review showed limited literature in the field of patient safety culture and incident documentation systems in Saudi Arabia [23–30]**.** Despite the large number of articles that endorse the significance of patient safety around the globe, this topic has not been adequately examined in Saudi Arabia. Therefore, the present study aimed to determine the challenges faced by clinicians in maintaining patient safety in EDs, in addition to assessing the barriers experienced by health professionals in using e-OVR in Saudi Arabia.

## Methods

### Study design and setting

A survey was conducted in the ED at King Khalid University Hospital (KKUH) in Riyadh, Saudi Arabia. The study was conducted between October 2017 and May 2019. The Institutional Review Board of the College of Medicine, King Saud University (KSU) approved the study. The healthcare professionals included in this study were all physicians and nurses working in the ED; they were selected using convenience sampling. These professionals signed the informed consent with a brief description of the study, including significance, and assured them of full confidentiality. Professionals who had less than three months’ experience in ED at KKUH were excluded. A self-administered questionnaire was distributed to 294 participants. Of these, 208 were returned, 11 of which were excluded due to incomplete information. In total, 197 completed questionnaires were used (response rate 67%).

### Questionnaire

An anonymous self-administered questionnaire was distributed either as a paper version personally by the researchers or as an electronic version by email. The questionnaire was adapted and modified from the Agency for Healthcare Research and Quality and from previously published research studies [31, 32]. The questionnaire was adapted in English only. The questionnaire consisted of a demographic section followed by six major sections: (1) work area/unit, (2) communication, (3) satisfaction and system usability, (4) system confidentiality and security, (5) workplace safety culture, and (6) training. Sections (1), (2), and (3) were pertinent to patient safety, whereas sections (4), (5), and (6) related directly to e-OVR. Sections (1), (3), and (4) were rated using a three-point Likert scale (1 = Agree, 2 = Disagree, 3 = Neither). Similarly, section 2 was rated using a three-point Likert scale (1 = Always or Often, 2 = Sometimes, 3 = Rarely or Never). Dichotomous variables such as yes or no were also used wherever applicable. The patient safety grade was measured as Excellent, Very Good, Acceptable, and Poor. The overall satisfaction with e-OVR was measured using a five-point Likert scale (1 = Very unsatisfied, 2 = Unsatisfied, 3 = Neutral, 4 = Satisfied, 5 = Very satisfied).

### Data analysis

The statistical package SPSS v.21 was used for data analysis. All parameters were summarized to compute frequencies, means, and percentages. For comparison between nurses and physicians’ categorical data, the chi-square test was used. Differences were considered statistically significant at *p* < 0.05.

### Results

A total of 197 participants, of which 48 (24%) were physicians and 149 (76%) were nurses were included in the analysis. The mean age of participants was 33 (7) years. Most of the participants were female (135, accounting for 68.5% of the study population). The majority (84.3%) of participants worked between 40 and 59 h per week. In terms of experience, 36% of participants showed between six- and ten-years’ experience in their profession. Table 1 shows the demographic characteristics of the participants.

**Table 1:** Demographics characteristics of nurses and physicians (*N* = 197)

Characteristics	Levels	n	%
**Gender**	*Male*	62	31.5
	*Female*	135	68.5
**Current profession**	*Physician*	48	24.4
	*Nurses*	149	75.6
**Duration in the current profession**	*Less than 1 year*	18	9.1
	*1 to 5 years*	43	21.8
	*6 to 10 years*	71	36.0
	*11 to 15 years*	36	18.3
	*More than 15 years*	29	14.7
**Working hours per week**	*Less than 20 h per week*	6	3.0
	*20 to 39 h per week*	21	10.7
	*40 to 59 h per week*	166	84.3
	*60 to 79 h per week*	3	1.5
	*80 or more*	1	0.5
	**Mean (SD)**		
**Age**	33.07 (7.44)		

### Patient safety concerning work area or unit

**Fig. 1:**
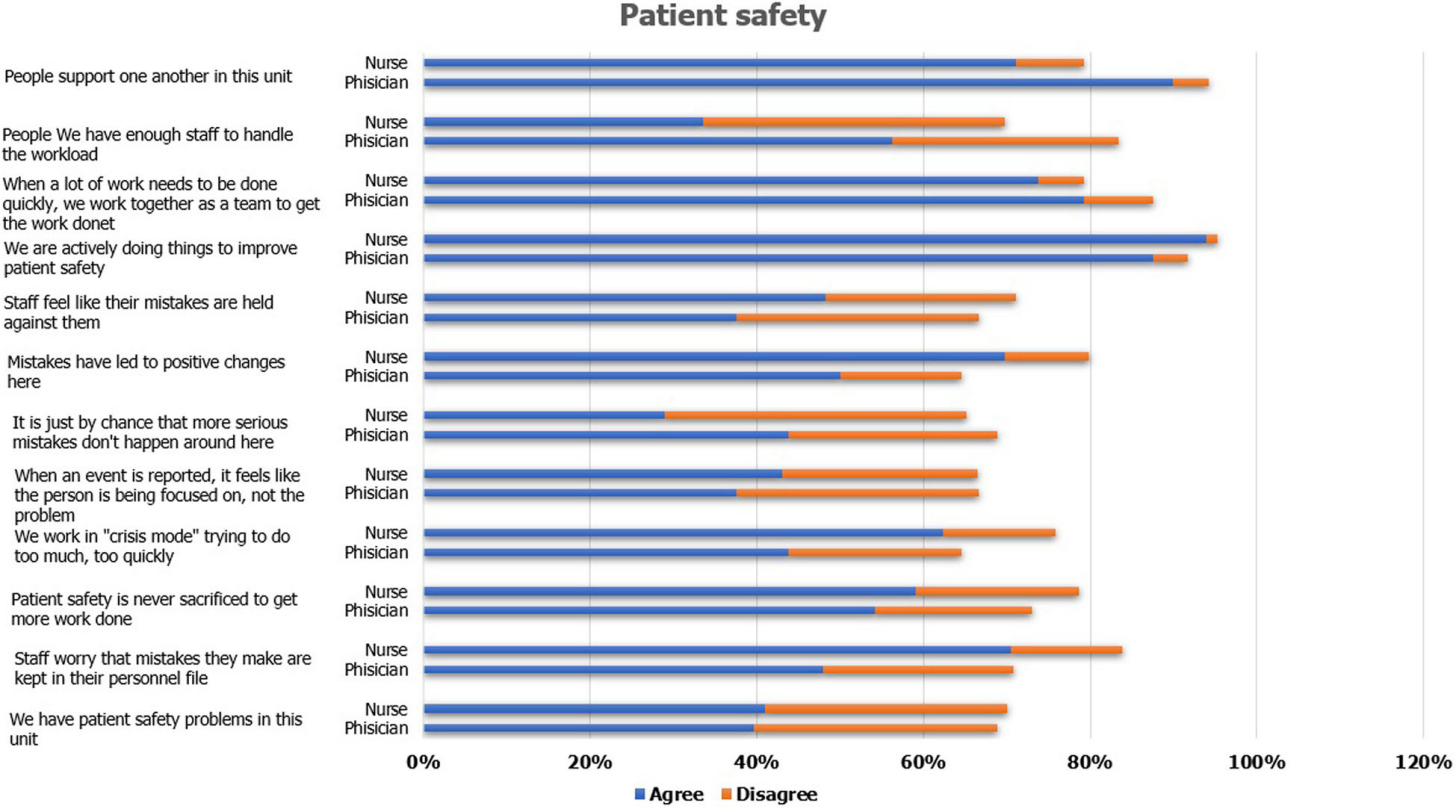
Patient safety concerning work area or unit

Here, 76% of participants agreed to support one another in the work area. Figure 1 shows the responses regarding patient safety related to the work area or unit. A third of the participants (34%) reported that they did not have enough staff to handle the workload between nurses and physicians (*p* = 0.017). Furthermore, more than half of the staff agreed that they worked in “crisis mode,” trying to do too much, too quickly (*p* = 0.075). Twenty-six percent of participants assented to the use of more temporary staff to improve patient care (*p* = 0.003). Approximately 92% expressed the view that they are actively doing things to improve patient safety. About 65% of participants believed that their mistakes are kept in their personnel file (*p* = 0.017). Moreover, after reporting an event, 42% of participants felt that they were focused on, rather than the problem. However, 65% of all participants assumed that their mistakes had led to positive changes during the process of emergency healthcare. Overall, most participants agreed that the system is good for preventing errors from happening between the two groups (*p* = 0.012).

### Patient safety and communication issues

**Fig. 2:**
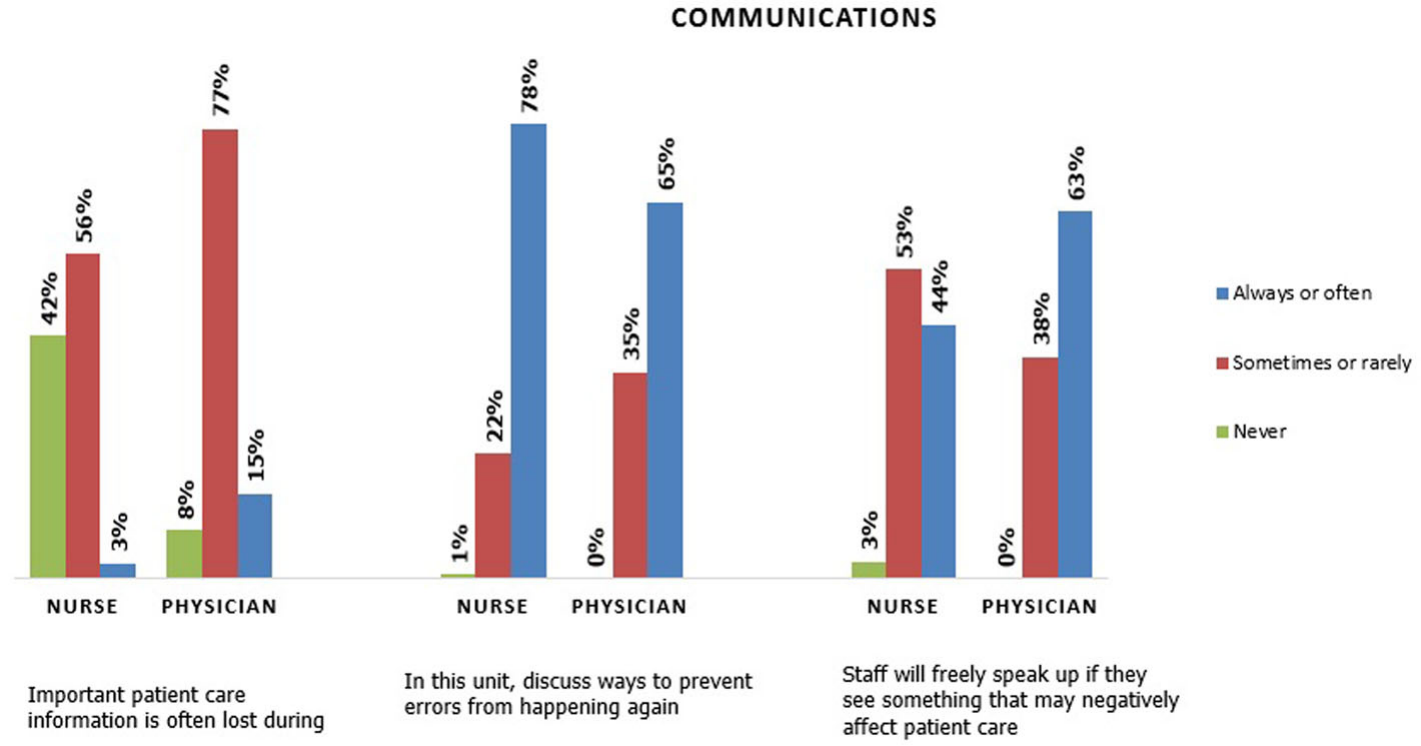
Patient safety and communication issues

The majority of staff (75%) discussed ways to prevent errors from happening again. However, when something that could negatively affect patient care arose, only half of the participants felt free to speak up. Moreover, the study found that 44% of participants rarely or never questioned the decisions or actions of those with more authority, with strong significance (*p* < 0.001) between nurses and physicians. Surprisingly, 61% of staff participants missed important patient care information during shift changes (*p* < 0.001). Sixty-seven percent of participants reported experiencing some sort of violence from patients, and productivity among more than half of them had been affected. Nearly 60% of the participants agreed that the 24-h access to hospital, along with the presence of drugs, made healthcare facilities a target of violence (*p* = 0.006). Figure 2 shows responses relating to patient safety and communication issues. However, 38% of participants graded the patient safety in the emergency department as “Very Good”, whereas 40% graded patient safety as “Acceptable”.

### Satisfaction and system usability of e-OVR

Table 2 shows our findings regarding e-OVR satisfaction, usability, training, confidentiality, and security. Almost 85% of the health professionals sampled knew where to access the e-OVR form and how to use it. Results showed that physicians have less knowledge regarding how to access (45.8%) e-OVR than nurses (98%; *p <* 0.001)*.* Only 44% of physicians knew how to use e-OVR compared to 94.6% of nurses (*p* < 0.001). The classifications, categories, and subcategories of the e-OVR system ‘Incident Reporting Form’ were found to be easy to understand and sufficient to report the incidents by nearly 75% of participants. Most of the participants (80%) agreed that reporting an incident on computer is easier than reporting an incident on paper. Again, physicians (54%) showed less agreement in comparison to nurses (89%; *p* < 0.001). Fifty-five percent of the staff stated that the reporting system is stable (*p* < 0.001). The process of reporting incidents through e-OVR produced variation in opinions among staff: 33% of total participants admitted the complexity of e-OVR (*p* = 0.28), 32% of both nurses and physicians found the reporting to be time consuming (*p* = 0.002) and 17% agreed that a lot of mental effort is required to report using e-OVR (*p* < 0.001). More than half of participants (58%) were able to determine the follow-up status for any incident that they had reported (*p* < 0.001).

**Table 2:** e-OVR satisfaction, usability, training, confidentiality, and security system

Characteristics	Levels	Nurses		Physicians		Total		*P* Value
		n	%	n	%	n	%	
**Satisfaction & System Usability**								
I know where to access the e-OVR from	Agree	146	98	22	45.8	168	85.3	0.000
	Disagree	2	1.3	22	45.8	24	12.2	
I understand how to use the e-OVR system.	Agree	141	94.6	21	43.8	162	82.2	0.000
	Disagree	2	1.3	22	45.8	24	12.2	
The e-OVR incident reporting process is complex.	Agree	50	33.6	14	29.2	64	32.5	0.282
	Disagree	58	38.9	15	31.3	73	37.1	
The e-OVR reporting process is time consuming	Agree	45	30.2	17	35.4	62	31.5	0.002
	Disagree	70	47	10	20.8	80	40.6	
Using the e-OVR system requires a lot of mental effort	Agree	26	17.4	9	18.8	35	17.8	0.000
	Disagree	84	56.4	12	25	96	48.7	
**System Confidentiality and Security**								
We have a non-punitive culture (no blame culture) of reporting in our hospital (i.e., punishment or discipline after reporting is not promoted).	Agree	75	50.3	21	43.8	96	48.7	0.529
	Disagree	31	20.8	9	18.8	40	20.3	
Reporting the incidents could improve the quality of healthcare & patient safety	Agree	131	87.9	42	87.5	173	87.8	0.935
	Disagree	2	1.3	1	2.1	3	1.5	
There is disciplinary action after reporting the incidents	Agree	89	59.7	10	20.8	99	50.3	0.000
	Disagree	15	10.1	7	14.6	22	11.2	
I can get timely feedback after reporting	Agree	76	51	5	10.4	81	41.1	0.000
	Disagree	27	18.1	16	33.3	43	21.8	
The culture in our hospital is: the higher the number of incident reports, the better	Agree	59	39.6	10	20.8	69	35	0.002
	Disagree	37	24.8	7	14.6	44	22.3	
**Training**								
The training provided included sufficient knowledge & skills	Agree	135	90.6	32	66.7	167	84.8	0.000
	Disagree	6	4	7	14.6	13	6.6	
I understand what should be reported	Agree	144	96.6	27	56.3	171	86.8	0.000
	Disagree	1	0.7	8	16.7	9	4.6	
I understand the importance of reporting incidents	Agree	144	96.6	38	79.2	182	92.4	0.000
	Disagree	1	0.7	3	6.3	4	2	
Have you ever reported an incident using electronic OVR system?	Yes	139	93.3	17	35.4	156	79.2	0.000
	No	10	6.7	31	64.6	41	20.8	

### System confidentiality, security, and training

The majority of the participants agreed that the security and privacy of the e-OVR system for staff and patients is adequate and significant (*p* < 0.001). The presence of a non-punitive culture in the ED was stated by approximately half the staff. On the other hand, 50% agreed that there were disciplinary actions after reporting (*p* < 0.001). However, 54% of staff believed that the e-OVR executive approached the reports systemically instead of individually (statistical significance found, *p* = 0.002). Approximately 70% of all incidents reported in the hospital are analyzed to determine causes, discussed in a departmental meeting, and result in a plan for improvement. However, 22% of the professionals did not receive timely feedback, with strong significance found (*p* < 0.001). Most of the participants (85%) reported that the training provided included sufficient knowledge and skills of e-OVR. Most respondents understood what should be reported (87%, *p <* 0.001) and the importance of reporting (92%, *p* < 0.001). Moreover, 79% of participants had reported at least one incident using e-OVR (*p* < 0.001) (see Table 2). In terms of overall satisfaction with the e-OVR system, 52.3% of nurses reported satisfaction, whereas 50% of physicians were neutral, which was found to be significant.

## Discussion

The present study was designed to address patient safety challenges and barriers to e-OVR use in the ED. Incident reporting is considered a core process to enhance the quality of the healthcare system and patient safety. Therefore, healthcare organizations must consider the prioritization of patient safety as an ethical obligation in the context of healthcare.

### Patient safety perceptions among participants

The current study findings support what previous studies found regarding high workload as a result of less staff [24, 29, 30]. Eventually, the shortage of professional employees will negatively affect patient care by staff experiencing burnout from being overworked and because of high stress [33, 34]. The personalization of mistakes and reported events could indicate the presence of a blame culture in our ED, which has been highlighted in the literature [24, 27–30]. Furthermore, a blame culture could affect patient safety and quality of care by prohibiting the willingness of staff to report incidents [28, 29, 35].

Communication is a key part of providing good quality patient care, and it reflects the work of staff as an organized team. Leonard et al. in 2004, stated that failure in communication is responsible for 70% of events reported to the Joint Commission for Hospital Accreditation [9]. Thus, encouraging staff to speak freely is the result of good communication between team members/units. Unfortunately, communication is a problem in our region, which is supported by other authors [24, 29, 30, 36]. In addition, our findings emphasize that information is lost during shift changes in the ED, which is a stressful environment due to ongoing changes and is supported by other studies [23, 30, 36]. This can be explained by the observation that overcrowding, communication issues, workload, and pressure in the ED are the challenges encountered during the process of care, possibly leading to loss of information [4–6, 37, 38].

This study revealed that two out of three participants had faced some sort of violence from patients. Violence towards nurses and physicians is unpredictable and burdens the healthcare system [39]. Violent behavior against healthcare workers has serious consequences for the professionals involved in the process of patient care. Several factors have been identified in previous studies that contribute to violence, such as patients’ frustration due to stress, verbal abuse, overload, fatigue, and pressure in healthcare professionals [40–42]. Nevertheless, person- related factors were identified in previous studies such as miscommunication, misunderstanding, rudeness, and failure to deliver information in an appropriate way to the patient or patient’s family [2, 9, 24, 40].

### E-OVR perceptions among participants

Several significant barriers that prevented staff from using e-OVR, including absence or delayed feedback, and the required forms being complex and requiring mental Effort, were observed in the current study. Similar factors have been reported by other studies [20, 43–45]. However, whereas our results showed that reporting by e-OVR is a time-consuming activity, other authors reported that lack of time among staff was one of the main drawbacks [43–45]. Moreover, fear of punishment and losing one’s job were reported in Saudi Arabia, while in Jordan fear of punishment was a barrier in reporting [17, 19, 46]. In terms of overall satisfaction with e-OVR, the results illustrated generally optimistic but mixed attitudes among participants. This study recommends that the administration of the hospital must understand the importance of regulatory requirements regarding e-OVR systems and assure staff of their responsibilities in reporting thus ensuring the correct response by the organization in a timely manner.

### Limitations

This study was performed in a single ED department; additional ED departments in other centers should be included in future studies. Although the current study focused on nurses and physicians, the majority of respondents in our study were nurses. In addition, the use of a convenient sampling technique limits the generalization of the results. Further studies are needed to examine factors other than those reported in this study. Despite these limitations, the study provides novel information that sheds light on several critical patient safety and e-OVR issues of importance to Saudi Arabian hospitals.

## Conclusion

Although patient safety is well emphasized in clinical practice, especially in the ED, many factors have been reported that may hinder patient safety, including staff shortages, missing vital data during shift changing, and violence towards clinicians. Violence is a major problem in EDs, and staff shortages may affect performance. Further efforts are needed to eliminate violence and to emphasize the needs of additional staff in the ED. On the other hand, reporting and documentation should be well supported by continuous staff training, help, and feedback.

## Data Availability

The datasets of the current study available from the corresponding author on reasonable request.

## Abbreviations

ED: Emergency department
e-OVR: Electronic occurrence variance reporting
HCP: Healthcare professional
KKUH: King Khalid University Hospital
